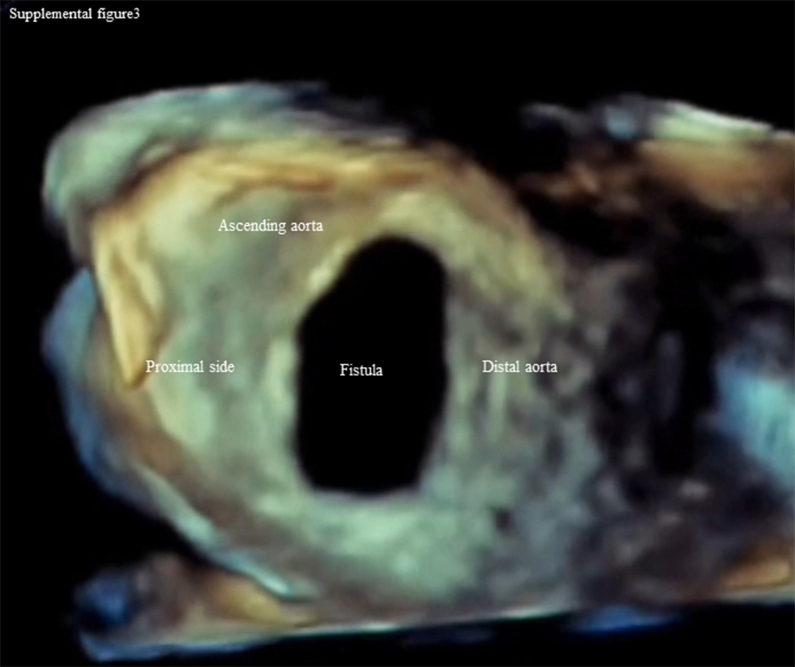# Rapidly Enlarged Ascending Aortic Pseudoaneurysm After Surgery

**DOI:** 10.1016/j.atssr.2024.06.012

**Published:** 2024-06-28

**Authors:** Hiromasa Nakamura, Yujiro Miura, Atsuyuki Mitsuishi, Keisuke Yoshida, Naoki Edo, Ren Saito, Kazumasa Orihashi

**Affiliations:** 1 Department of Cardiovascular Surgery, Kochi Medical School, Kochi, Japan; 2 Liaison Healthcare Engineering Section, Kochi Medical School, Kochi, Japan

Ascending aortic pseudoaneurysms are rare entities that can occur secondary to trauma, infection, connective tissue disorders, vasculitis, or as a complication of cardiac surgery.

In the present case, methicillin-resistant *Staphylococcus aureus* bacteremia was thought to have broken down the cardioplegia injection site of previous operation, forming a rapid pseudoaneurysm of the aorta.

The patient is an 85-year-old man who had a pericardiectomy 8 months earlier, followed by omentopexy due to mediastinitis. Thereafter, methicillin-resistant *Staphylococcus aureus* bacteremia persisted, and long-term antibiotic therapy was being administered.

A computed tomography 20 days before onset of disease showed no obvious pseudoaneurysm formation (Supplemental Figure 1). However, a pseudoaneurysm rapidly formed thereafter, and its size was measured to be a 36 × 60 mm pseudoaneurysm with a 31-mm fistula (Figure 1, Supplemental Figure 2). Transesophageal echocardiography indicated the presence of a pseudoaneurysm with a fistula (Figure 2, Supplemental Figure 3). Figure 3 shows intraoperative findings. A fistula was detected in the ascending aorta.

**Figure 1:**
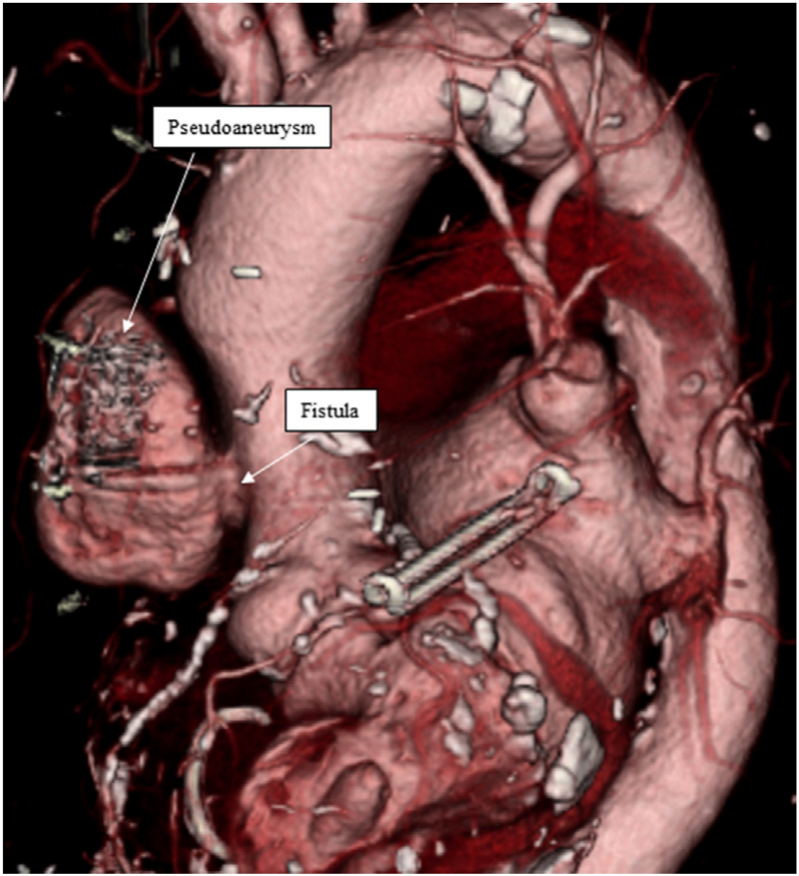

**Figure 2:**
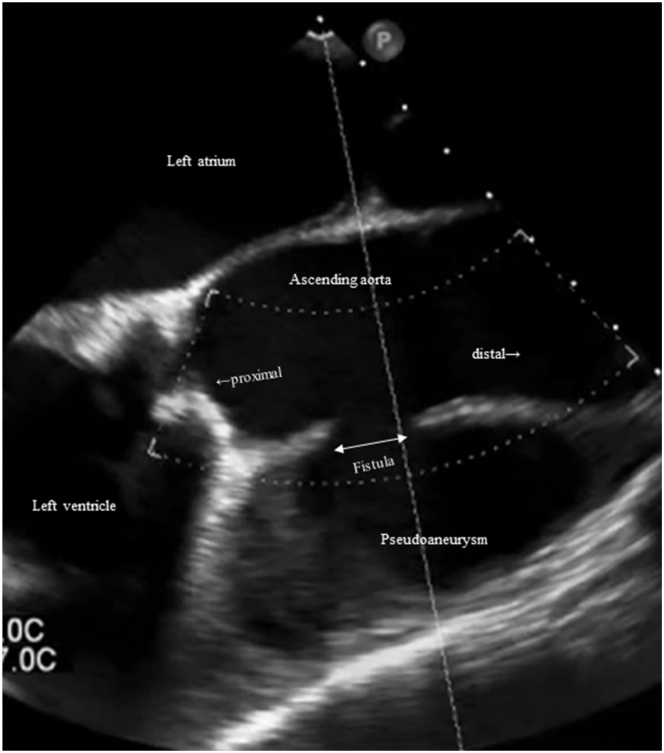

**Figure 3:**
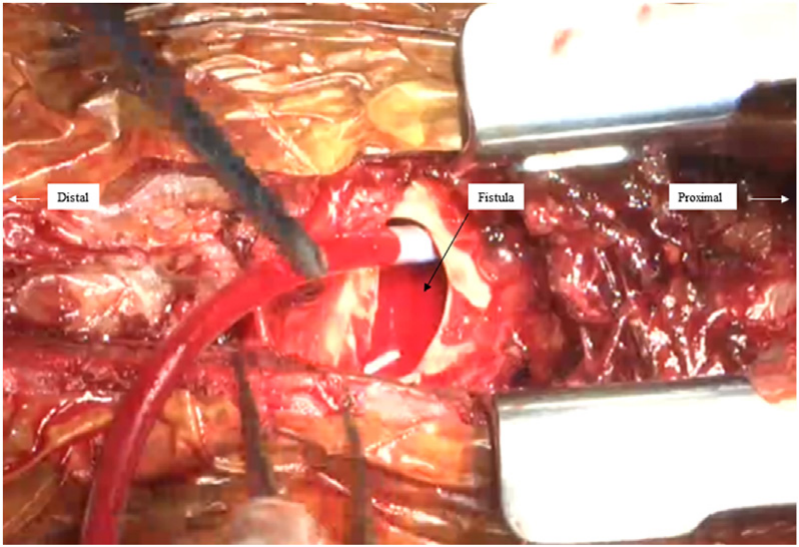

## Figures and Tables

**Supplemental Figure 1:**
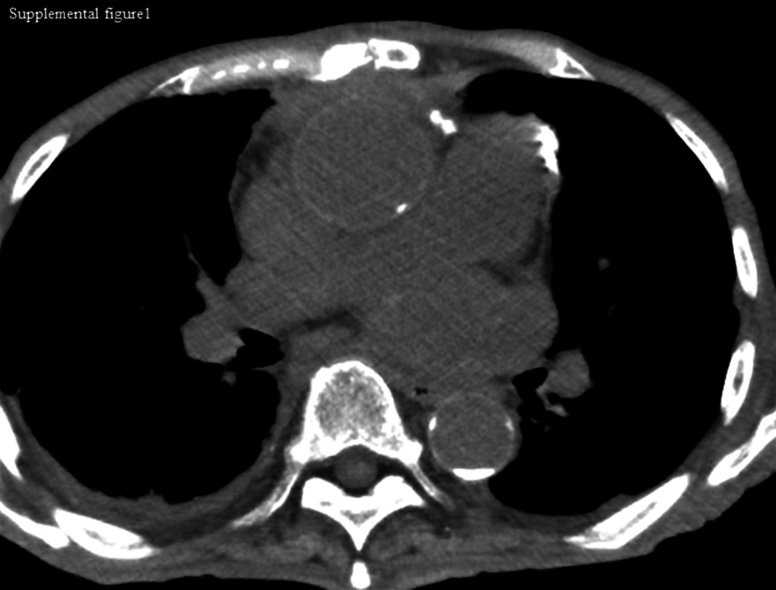


**Supplemental Figure 2:**
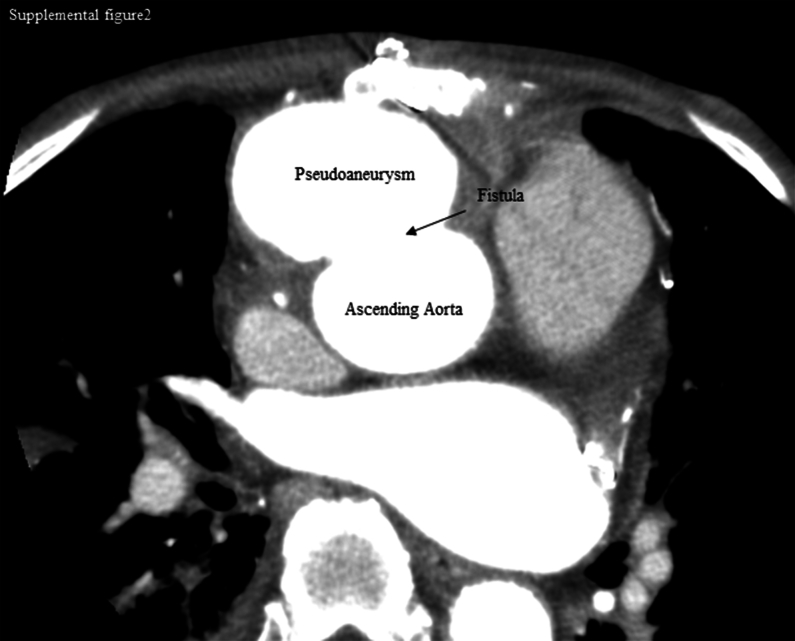


**Supplemental Figure 3:**